# Health of Residents

**Published:** 1907-04-27

**Authors:** 


### Resident Medical Officers' Department.

### HEALTH OF RESIDENTS.

When one considers the arduous and responsible duties and the scanty leisure of a house surgeon's life in most large hospitals, the wonder is, not that some fall sick and have to resign, but that any complete their appointments. A year's hard work in hospital is a fairly severe test, even of the strongest constitution, and it is remarkable how many men of only moderate physique come through this ordeal without damage to their health. The reason must lie in the fact that congenial labour, however onerous and exacting, is seldom harmful, so long as it is varied and interesting. Fortunately, most hospital residents are really devoted to their work, and find keen enjoyment in the skilful performance of even the dullest and most distasteful of their duties. Were it otherwise, there would doubtless be many more cases of mental and physical breakdown among house officers than there are at present.

A newly-qualified practitioner, before entering upon his duties as a house surgeon, would do well to read and take to heart, the admirable advice which is given in the Introduction to Heath's "Minor Surgery," on the subject of the care of his own health during his life in hospital. Written many years ago by the late Christopher Heath, these few sentences are just as true and as pertinent at the present day as they ever were; and, in spite of the changes and elaborations in the duties of a house surgeon, the advice given is still practicable.

An adequate supply of good food to the medical officers' table is essential, if their work is to be done well. This is a point which hospital committees have begun to realise. The food should also be attractively served, and here the committees are not always so considerate: they do not all seem to understand that the appetite must be tempted when the work, if not exactly sedentary, is, at any rate, performed in close wards and stuffy out-patient rooms, amidst sights and smells which are not calculated to give a relish to an ill-served, even if perfectly wholesome, meal.

The relief of the sick poor has naturally the first claim upon the resources of a hospital, and the strictest economy in the administration of the charity is, of course, expected of its governors. But economy need not be carried to its extreme limits at the dining-table of the resident staff. Interrupted meals are often unavoidable; but the fare need none the less be appetising.

Fresh air in anything like sufficient quantity is difficult for a house surgeon to obtain. But, except when he is "taking in" or "on duty," it is usually possible for him to slip off every day for at least an hour's walk on the Embankment or in one of the parks; and on Sundays a longer outing is generally within his reach, if he makes up his mind to get it. Even an hour in the open air is better than nothing, and when it is regularly taken the advantage to health and spirits is very great. The open bedroom-window and the morning cold bath need not be insisted on here, for no sane house officer nowadays neglects these obvious duties, but we might suggest that a few minutes with dumb-bells or a "developer" before the bath are an excellent preparation for a day's hospital work, when other exercise is out of the question.

It must be owned that to seize every small opportunity for fresh air and exercise needs some determination. It is, no doubt, easier to spend a spare hour with a book and a pipe in one's room rather than to make arrangements with a colleague and go for a walk or a bicycle ride, or even for an airing on a motor 'bus; and there is some truth in the objection that an ordinary day's work is usually a sufficient test of the will-power without exercising it in off-duty hours. But the resulting gain is worth the effort.

Night work is a trial of nerves and health which cannot be avoided, and it often happens that the most broken nights follow the hardest days. The man who can take a little or much sleep when and where he chooses is as rare as he is fortunate. To most men a thrice disturbed night means a night without any sleep at all, and a miserable day to follow. Happily, much night-work at a stretch is not common in hospital. Even if he anticipates being called back to the wards during the night, the resident should go to bed immediately after his night round, and should enjoy as much sleep as he can before the summons comes. It is sheer foolishness to sit in a chair fully dressed, perhaps for hours, with aching eyes and weary limbs, consuming tobacco and whisky long after the pleasure has gone out of them, just because "it isn't worth while going to bed when you're sure to be knocked up as soon as you're asleep."

Night work is, indeed, the worst part of a doctor's life, and in hospital it is just as trying as in private practice. For although the general practitioner has to dress more fully and to go longer distances, the house surgeon is often sent for because he is at hand and is bound to come, although it is clear he can do no good, when—under like circumstances—the country doctor would usually be spared.

An occasional week-end visit to the country, in addition to the summer holiday (which, it is to be hoped, is always insisted upon), is a great relief from the strain of hospital life. It is just long enough to break the monotony of work and to fill the lungs with fresh air, and just short enough to avoid the unsettling effect of an ordinary holiday. Its good effect is often quite remarkable.

Many house surgeons at the outset of their appointment promise themselves that in their spare evening hours indoors they will systematically read up their cases, or study for the higher examinations. Very few, however, succeed in keeping these promises, except in the most desultory way. It is, indeed, doubtful whether most men are not better employed after a hard day's professional work in relaxing their minds rather than in spurring them on to further efforts of the same sort. We admit that a certain amount of reading up of cases is essential for the good of their patients and the better education of themselves. But all work and no play makes dull house surgeons, and a couple of rubbers of bridge, or a game of billiards, is a better preparation for a good night's sleep than is an hour spent in feverish efforts at reading pathology for the final F.R.C.S. Certain hospital appointments are notoriously easier than others, and the holders of these would indeed be foolish if they did not use their ample leisure hours for the purpose of professional study; but the foregoing advice applies to the majority of resident medical officers, and especially to house surgeons, whose work is usually the most exacting of all.

It is a common procedure for the more ambitious men to take successive house appointments, sometimes at the same institution, more commonly at different hospitals, and thus to obtain a large and varied experience. We would urge all but the strongest of these to see to it that an interval elapses between each appointment, even if this means the loss of an attractive post. A short voyage as ship's surgeon provides an ideal holiday after a year's work in hospital; and the time thus spent is not wasted, for much reading can, if necessary, be done on board ship, and the opportunity for travelling and seeing something of foreign lands is one which may quite possibly not occur again for many years.

We have now outlined the house surgeon's duty towards himself, and we would conclude by reminding him that without due attention to his own health he will sooner or later be unable to perform his duty towards his patients.

